# Pleomorphic Xanthoastrocytoma of the Frontal Lobe in a Child: A Rare Entity

**DOI:** 10.7759/cureus.15566

**Published:** 2021-06-10

**Authors:** Leopoldo Mandic Ferreira Furtado, José Aloysio Da Costa Val Filho, Gustavo Alberto Rodrigues da Costa, Patrícia Salomé Gouvea Braga

**Affiliations:** 1 Department of Pediatric Neurosurgery, Vila da Serra Hospital, Nova Lima, BRA; 2 Department of Pathology, Biocor Institute, Nova Lima, BRA

**Keywords:** neuro oncology, pleomorphic xanthoastrocytoma, surgery, brain neoplasm, frontal lobe

## Abstract

Pleomorphic xanthoastrocytoma (PXA) is an infrequent neoplasm that affects children less commonly than adults. In this case report, a four-year-old boy presented with focal seizures has diagnosed with this tumor in the frontal lobe. Complete surgical resection was achieved, and histopathological features of PXA grade II were observed. During follow-up, the patient showed improvement of the focal seizures. In spite of the pleomorphic features, the PXA had a favorable prognosis.

## Introduction

Pleomorphic xanthoastrocytoma (PXA) is a low-grade glial neoplasm originates from subpial astrocytes and first described at the end of the seventies [1-3]. In addition, PXA could present frequently as a grade II tumor which dissemination through cerebrospinal fluid has been reported a few times [4].

PXA could arise in many locations of the central nervous system (CNS), such as the temporal, parietal, and frontal lobes; cerebellum; pineal region; and spinal cord [5]. According to Mallick et al. [6], the temporal lobe is affected in almost half of all the cases. Although several reports in the literature have described the histopathological and genetic features of the tumor, no report has focused on the surgical aspects so far [1-4,6-10].

The goal of this case report was to elucidate the overall characteristics of PXA in order to provide a differential diagnosis and present a literature review.

## Case presentation

**Figure 1:**
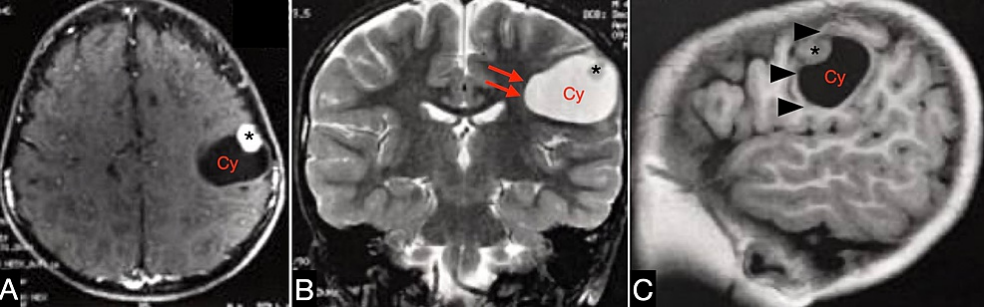
Imaging features of pleomorphic xanthoastrocytoma. The axial-weighted magnetic resonance imaging (MRI) scan after the addition of gadolinium shows a nonenhanced cystic lesion with an intensely enhanced mural nodule (*) in the left frontal lobe (A). The T2-weighted MRI scan in the coronal view shows the hyperintense cystic lesion (Cy) with an isointense mural nodule that led to a discrete mass effect in the surrounding frontal lobe (double red arrows) (B). The T1-weighted sagittal MRI scan shows the tumor occupying the postcentral gyri (triple arrowheads) on the left frontal lobe (C).

A four-year-old boy first presented with epileptic focal seizures in the right hemiface without generalization for three months. Care of the patient was primarily provided by a neurologist, who initiated carbamazepine therapy, which achieved a satisfactory control of the patient’s condition. In addition, the patient had no compromised physiological milestones and had normal neurocognitive developmental and regular neurological examination results. Brain imaging was performed, and magnetic resonance imaging displayed a tumor in the left frontal lobe which depicted a heterogeneous shape, with solid and cystic portions (Figure 1).

Thus, surgical resection was indicated.

For the surgical technique, the patient was placed in a prone position, with his head fixed with pins. Neurophysiological intraoperative monitoring was conducted owing to the close proximity of the tumor to the precentral gyri. The somatosensory-evoked potential monitoring and brain mapping were performed during surgery. Left frontoparietal craniotomy was performed, and after the dura was opened and directed toward the superior sagittal sinus, a small corticectomy was performed in the superior frontal sulcus. Macroscopically, the solid part of the tumor had a remarkably thick consistency, and the cystic portion was completely occupied by a translucid fluid. The cyst was surrounded with a thick capsule.

As complete resection was achieved, hemostasis was performed, and the bone flap was fixed using absorbable plates and screws. The patient demonstrated an uneventful postoperative period.

**Figure 2:**
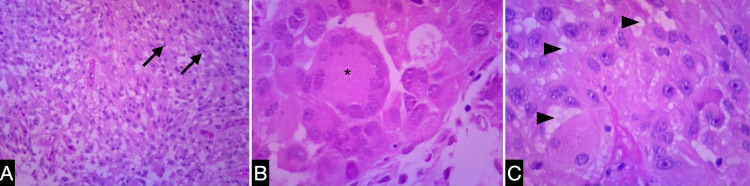
Histopathological features of pleomorphic xanthoastrocytoma. The histopathological findings include irregular spindle-shaped and ovoid cells (black arrows) in a fibrillary background (A). Occasional multinuclear giant cells (*) (B) and xanthomatous cells with many cytoplasmic vacuoles (arrowheads) (C).

The tumor sample depicted marked heterogeneity and was formed by ovoid and multinuclear giant cells. In addition, some areas featured cytoplasmic inclusions and high degrees of vascularization. Therefore, the pathology was consistent with PXA grade II (Figure 2).

The patient showed adequate control of seizures. At four months after hospital discharge, the anticonvulsant was removed from the treatment regimen. No adjuvant treatment was needed, and the tumor did not recidivate.

## Discussion

In this case report, we describe a PXA occupying the frontal lobe in a child who presented with focal seizures. Although cases are infrequent in the young age group, such neoplasm could be diagnosed in a wide range of ages and are responsible for less than one percent of all brain tumors [9]. Furthermore, seizures in general are the most common presentation, and even musical hallucination was reported [11]. Concerning the anatomical distribution, the frontal lobe is affected in the third position of supratentorial sites, and the rarest cases were described as infratentorial [6].

The imaging feature observed in the present case report was a solid mass surrounded by a cystic portion with a mass effect and no hemorrhage signs. The heterogeneous appearance with peripheral contrast enhancement led us to consider other differential diagnoses such as primitive neuroectodermal tumors, pilocytic astrocytoma, atypical teratoid rhabdoid tumors, and glioblastoma [12,13]. Nevertheless, most reports recognized the mural nodule with a cystic lesion as a remarkable imaging finding in children and adults. Some cases with multiple cysts were also observed [9].

Surgical resection remains the cornerstone of treatment in which combination with a low tumor mitotic rate would improve the overall outcome. In addition, anatomically, this tumor presents more superficially in the brain, allowing for an easier surgical approach [9]. On the other hand, some reports indicated that tumor hemorrhage could translate into more difficulty intraoperatively, and which would be especially dangerous in children with low weights. Although hemorrhage has been considered a rare characteristic of this tumor, Crespo-Rodriguez et al. [14] reported hemorrhage occurring in 33% of their cases.

Concerning adjuvant therapy, the best evidence does not support the use of adjuvant radiotherapy. In a recent meta-analysis, 17.6% of 167 patients received this treatment, but this proportion was considered insufficient to obtain evidence. Therefore, the authors concluded that both radiotherapy and chemotherapy are not recommended for PXA [6]. Conversely, anaplastic PXA deserves a complementary therapy [7,11]. However, according to the latest WHO classification of 2016 [15], PXA and anaplastic PXA were considered as distinct entities.

Microscopically, this lesion presented moderated cellularity, predominantly pleomorphic tumor with no necrosis and rare mitoses, and occasional presence of bizarre multinucleate cells [16,17]. In spite of this appearance, the tumor presents a favorable prognosis, and the overall survival was 209 months [6]. Currently, the molecular definition of BRAF or wild-type mutation has prognostic importance, and BRAF positive patients had a better outcome than wild-type mutation [17].

## Conclusions

PXA is a rare brain tumor for which maximum operative resection is the mainstay for achieving the best outcome owing to the lack of evidence of the effectiveness of adjuvant radiotherapy and chemotherapy.
